# Supplementary material for: Single Ultra-High Dose Rate Proton Transmission Beam for Whole Breast FLASH-Irradiation: Quantification of FLASH-Dose and Relation with Beam Parameters
Source: Cancers (Basel). 2023 Apr 30;15(9):2579. doi: 10.3390/cancers15092579 (PMC10177419; doi:10.3390/cancers15092579)
Supplement: Supplementary file 1 [file cancers-15-02579-s001.zip › cancers-2348825-supplementary.pdf]

Figure S1: (A) dose-volume histogram of the PTV (red) and body (green) for the plans with varying minMU (300-1000). PTV DVHs for minMU=700-1000 show increasing coverage, while for 400-700 minMU DVHs are the same with the best target coverage; (B) average absolute FLASH-dose (Gy) for the different minMU-plans and machine settings. The bars in red correspond to an EB GC and those in blue to an SB GC; the darkest bars correspond to a minST=2ms, the medium-dark bars to minST=1ms, and the lightest bars to minST=0.5ms; for each minMU-plan the results are given for all three fractionation doses (5.7Gy, 9.7Gy and 14.3Gy) and per fractionation scheme the FLASH-dose is given for a maxN=200nA (left), 400nA (middle) and 800nA (right). All these results use the PBS-DR with a threshold of 1cGy and assume FLASH-thresholds of TD=4Gy,TDR=40Gy/s.

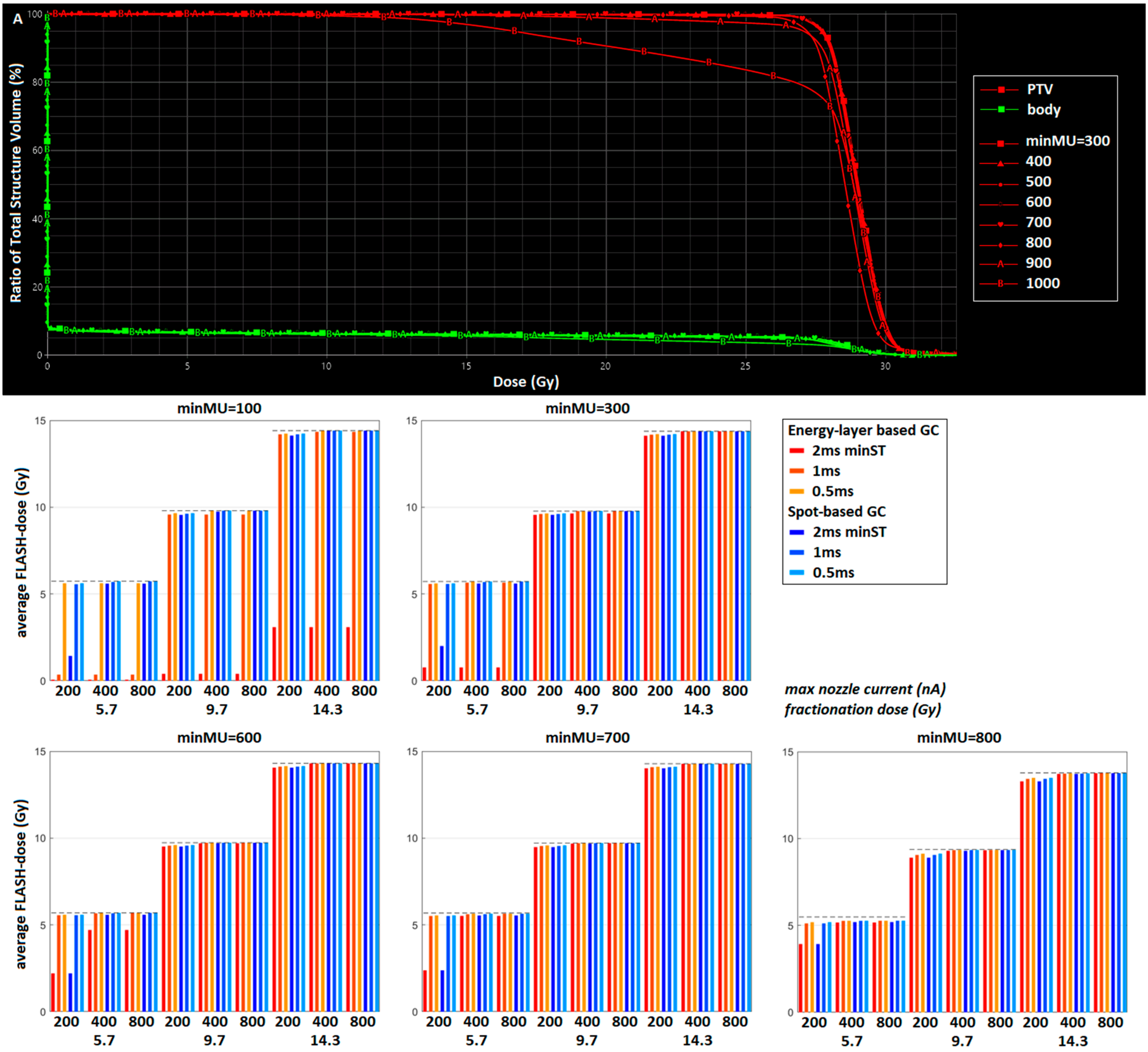

Figure S2: average FLASH-dose (% of total dose) for the different splitMU-plans and machine settings. The bars in red correspond to an EB GC and those in blue to an SB GC; the darkest bars correspond to a minST=2ms, the medium-dark bars to minST=1ms, and the lightest bars to minST=0.5ms; for each splitMU-plan the results are given for all three fractionation doses (5.7Gy, 9.7Gy and 14.3Gy) and per fractionation scheme the FLASH-dose is given for a maxN=200nA (left), 400nA (middle) and 800nA (right). All these results use the PBS-DR with a threshold of 1cGy and assume FLASH-thresholds of TD=4Gy,TDR=40Gy/s.

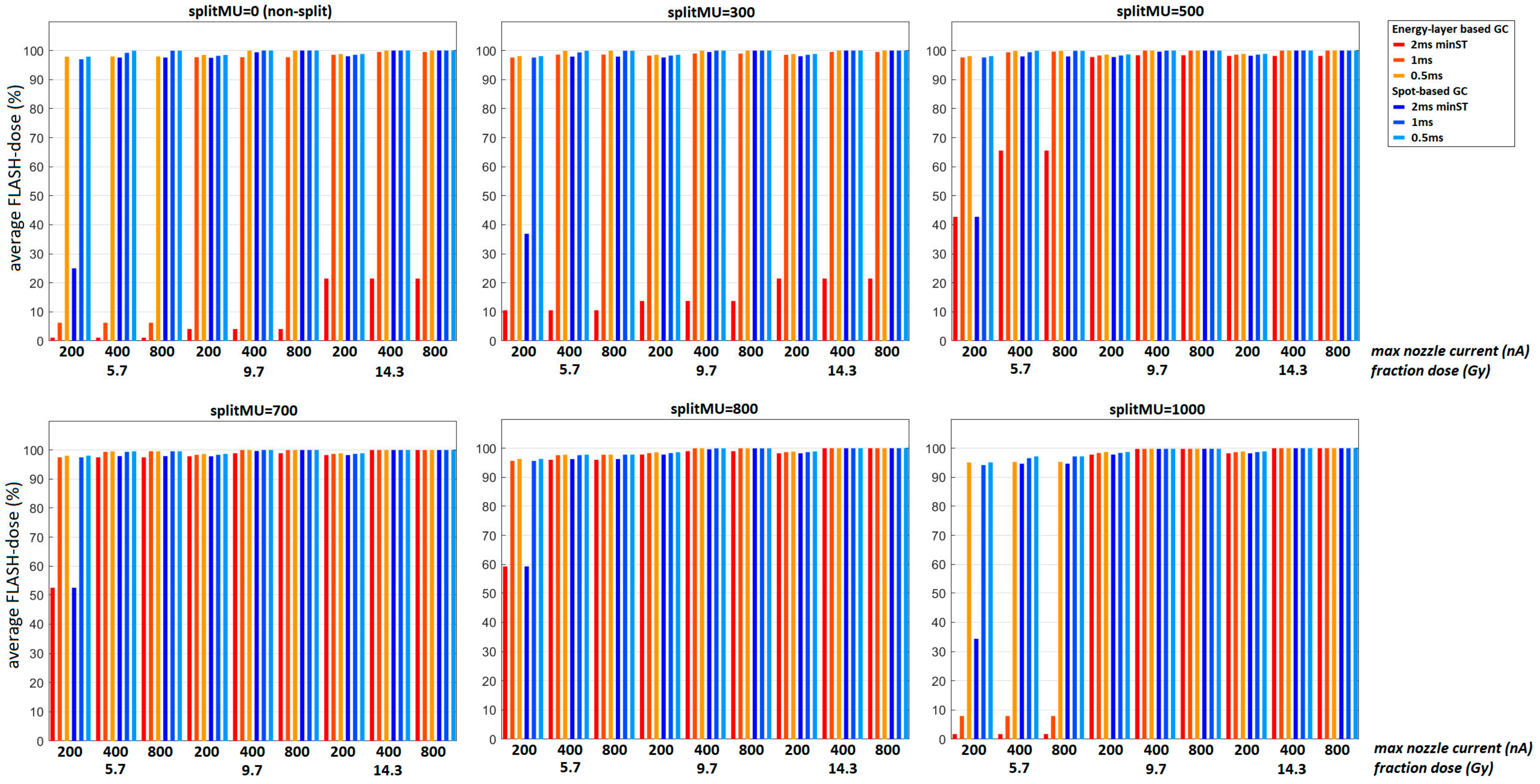

Figure S3: average FLASH-dose (% of total dose) for the split plan (minMU=100, splitMU=700) of the test case and three clinical cases using (A,C) the PBS-DR with a threshold of 1cGy and (B,D) the ‘sliding window’ method. (A,B) correspond to a FLASH dose-rate threshold (TDR) of 40Gy/s, (C,D) to TDR=100Gy/s; the FLASH dose threshold is 4Gy for (A-D). The bars in red correspond to an EB GC and those in blue to an SB GC; the darkest bars correspond to a minST=2ms, the medium-dark bars to minST=1ms, and the lightest bars to minST=0.5ms; for each case the results are given for all three fractionation doses (5.7Gy, 9.7Gy and 14.3Gy) and per fractionation scheme the FLASH-dose is given for a maxN=200nA (left), 400nA (middle) and 800nA (right).

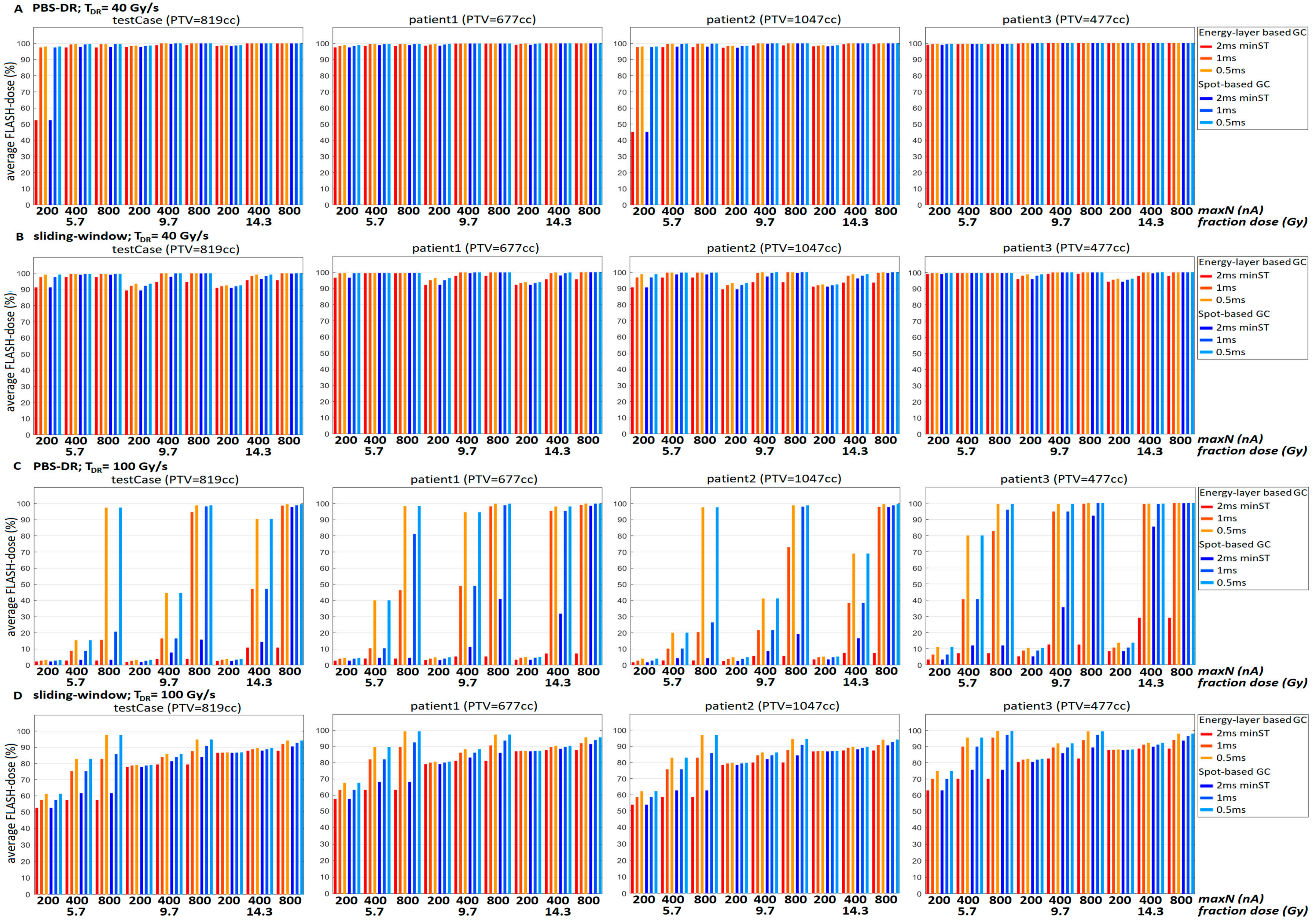

A PBS-DR; T_{DR}= 40 Gy/s
B sliding-window; T_{DR}= 40 Gy/s
C PBS-DR; T_{DR}= 100 Gy/s
D sliding-window; T_{DR}= 100 Gy/s
testCase (PTV=819cc)
patient1 (PTV=677cc)
patient2 (PTV=1047cc)
patient3 (PTV=477cc)
average FLASH-dose (%)
Energy-layer based GC
2ms minST
1ms
0.5ms
Spot-based GC
2ms minST
1ms
0.5ms
maxN (nA)
fraction dose (Gy)
200 400 800 200 400 800 200 400 800
5.7 9.7 14.3

Figure S4: average FLASH-dose (% of total dose) for the split plan (minMU=100, splitMU=700) of the test case and three clinical cases using the PBS-DR with a threshold of (A) 1cGy and (B) 10cGy. The bars in red correspond to an EB GC and those in blue to an SB GC; the darkest bars correspond to a minST=2ms, the medium-dark bars to minST=1ms, and the lightest bars to minST=0.5ms; for each case the results are given for all three fractionation doses (5.7Gy, 9.7Gy and 14.3Gy) and per fractionation scheme the FLASH-dose is given for a maxN=200nA (left), 400nA (middle) and 800nA (right).

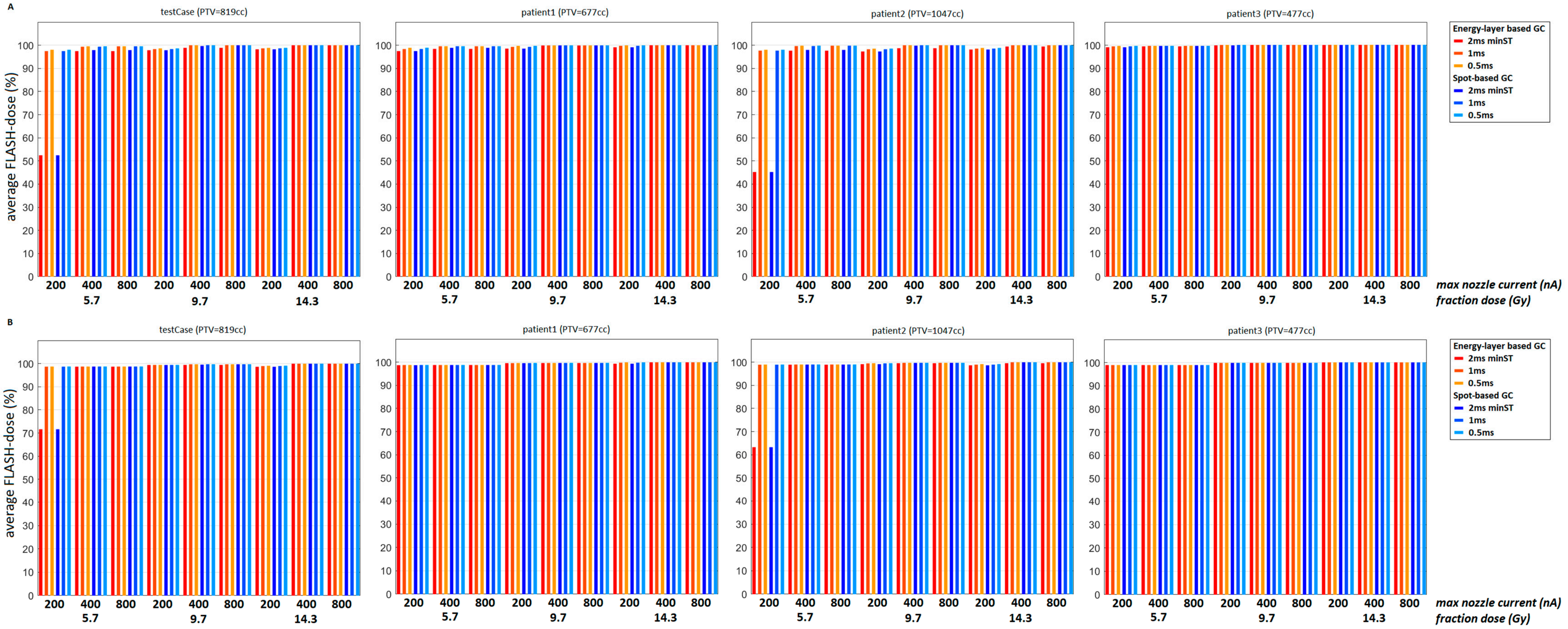

Figure S5: the dose contribution (Gy) in time (s) in a voxel for (A) a fraction of the 5x5.7Gy scheme and (B) the single fraction of 14.3Gy.

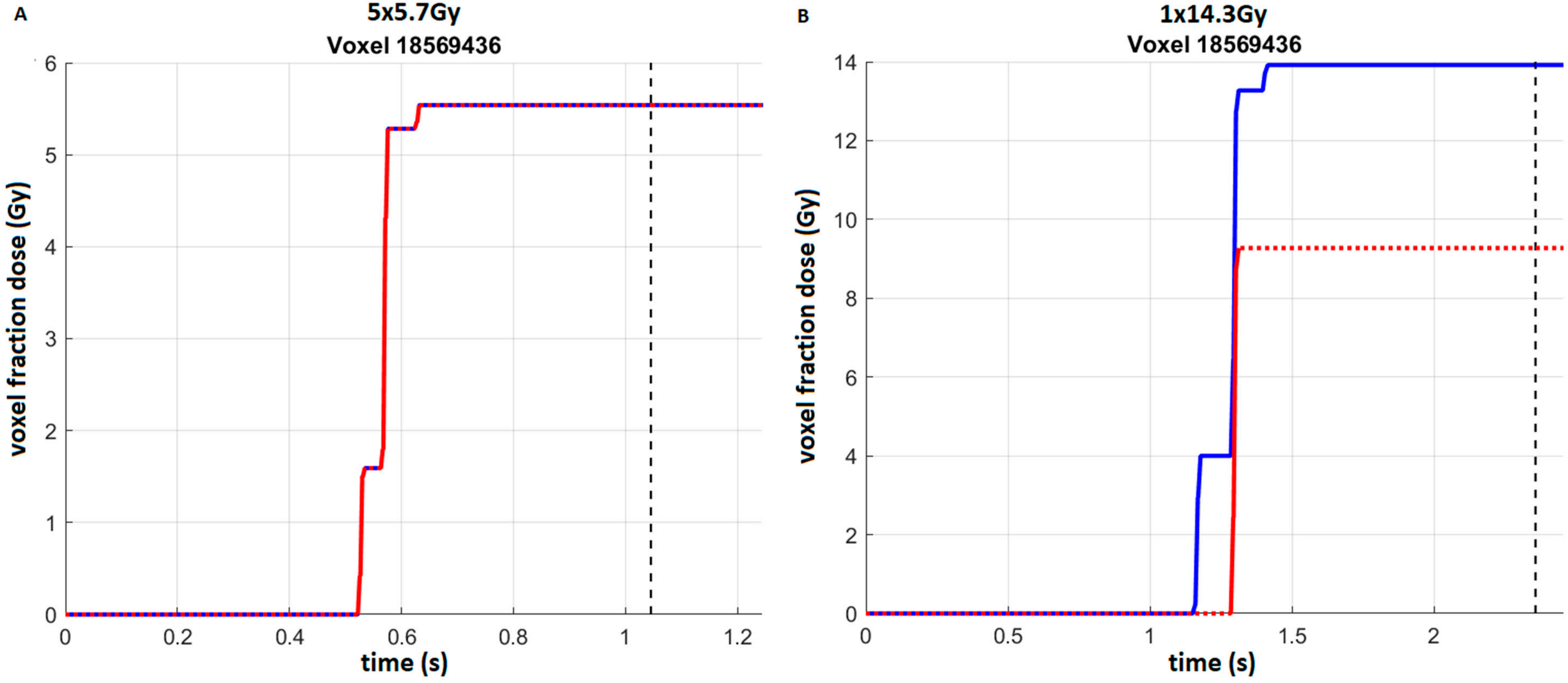

Table S1: table summarizing the assumed FLASH parameters, based on the template provided by Rothwell et al[50].

**Parameter**	**Value**
Dose threshold	4Gy
Dose-rate threshold	40Gy/s (and 100Gy/s for comparison two dose-rate calculation methods)
Dose-rate definition	PBS-DR using a 1cGy threshold; 'sliding window method'
Scanning speed/direction	10mm/ms; along smallest target dimension in beam's eye view
Minimum spot time	2ms (clinically possible); 1ms; 0.5ms (both technically feasible)
Maximum nozzle current	200nA (clinically possible); 400nA (technically feasible); 800nA (included to determine its effect on FLASH-dose)
Gantry current setting	Energy-layer based; spot based
Modality	Protons; pencil-beam spot scanning; transmission beam
Fractionation	5x5.7Gy; 2x9.74Gy; 1x14.32Gy
Number of beams	1 (or after plan splitting: 2, of which one is a "FLASH-beam" and the other a low-dose beam at non-FLASH DRs)
